# FRAILTY IN REACH II CAREGIVERS: A SECONDARY DATA ANALYSIS

**DOI:** 10.1093/geroni/igz038.1070

**Published:** 2019-11-08

**Authors:** Jeananne Elkins, Patricia C Griffiths

**Affiliations:** 1 Brenau University, Gainesville, Georgia, United States; 2 Emory University School of Medicine, Atlanta, Georgia, United States

## Abstract

Frailty, a reduction in reserve capacity in people who are otherwise considered healthy, affects between 9 and 13% of adults who are older. Frailty is a poorly understood syndrome; however, frailty is correlated with negative CV procedure outcomes, falls and institutionalization. Little is known about frailty in caregivers. A secondary data analysis was conducted using the REACH II publicly available dataset and the Groningen Frailty Index (GFI). At consent two percent of REACH II caregivers had difficulty going to the toilet while 11% had difficulty walking outdoors. More than 1/3 had hearing and vision losses. 75% felt sad or dejected. 82% were taking more than 4 medications. Based on their calculated GFI, between 61% and 64% of the REACH II caregivers were frail. Frail caregivers and their care recipient were less likely to go to the emergency department (-0.110 coefficient; p = 0.004 95% CI -0.184 -0.035) and were less likely to be hospitalized overnight during the past 6 months (-0.121 coefficient; p=0.004; 95% CI -0.203 -0.040). Frailty is an under-recognized syndrome in caregivers. Little is known about the impact of frailty on the caregiving dyad; however, ED utilization and hospitalization was decreased in these caregivers and their care recipients. This decrease may imply a delay in seeking care; and, in fact, lead to worse health outcomes for the dyad. With the aging of Baby Boomers and the continued dependence for long term care delivered by unpaid caregivers, implementation of programs to prevent and treat frailty in caregivers is essential.

